# Fish-Pak: Fish species dataset from Pakistan for visual features based classification

**DOI:** 10.1016/j.dib.2019.104565

**Published:** 2019-10-04

**Authors:** Syed Zakir Hussain Shah, Hafiz Tayyab Rauf, Muhammad IkramUllah, Malik Shahzaib Khalid, Muhammad Farooq, Mahroze Fatima, Syed Ahmad Chan Bukhari

**Affiliations:** a Department of Zoology, University of Gujrat, Pakistan; b Department of Computer Science, University of Gujrat, Pakistan; c Department of Fisheries and Aquaculture, University of Veterinary and Animal Sciences, Lahore, Pakistan; d Division of Computer Science, Mathematics and Science, Collins College of Professional Studies, St. John's University, New York, USA

**Keywords:** Fish species classification, Fish species recognition, Fish feature extraction, Fish scale, Fish head, Fish species shape

## Abstract

Fishes are most diverse group of vertebrates with more than 33000 species. These are identified based on several visual characters including their shape, color and head. It is difficult for the common people to directly identify the fish species found in the market. Classifying fish species from images based on visual characteristics using computer vision and machine learning techniques is an interesting problem for the researchers. However, the classifier's performance depends upon quality of image dataset on which it has been trained. An imagery dataset is needed to examine the classification and recognition algorithms. This article exhibits Fish-Pak: an image dataset of 6 different fish species, captured by a single camera from different pools located nearby the Head Qadirabad, Chenab River in Punjab, Pakistan. The dataset Fish-Pak are quite useful to compare various factors of classifiers such as learning rate, momentum and their impact on the overall performance. Convolutional Neural Network (CNN) is one of the most widely used architectures for image classification based on visual features. Six data classes i.e. *Ctenopharyngodon idella* (Grass carp), *Cyprinus carpio* (Common carp), Cirrhinus mrigala (Mori), *Labeo rohita* (Rohu), *Hypophthalmichthys molitrix* (Silver carp), and Catla (Thala), with a different number of images, have been included in the dataset. Fish species are captured by one camera to ensure the fair environment to all data. Fish-Pak is hosted by the Zoology Lab under the mutual affiliation of the Department of Computer Science and the Department of Zoology, University of Gujrat, Gujrat, Pakistan.

**Table:** Specifications Table

Subject area	Computer Science
More specific subject area	Image processing, Image identification, Image classification, computer vision
Type of data	Images
How data was acquired	A digital camera (Canon EOS 1300D) with a sensor type of CMOS bearing the resolution of 5202 × 3465 (Mpix) was used to acquired data.
Data format	JPG, Raw
Experimental factors	No such sample pre-treatment was conducted. However blue back ground color were considered instead of transparent and the Camera illuminance were ensured constant for each image at the time of capturing images.
Experimental features	The attributes of the subjects includes head features (mouth, snout, lips), body features (Elongated, streamlined, compressed), and the scale features (Dorsal fin, Pectoral fin, Pelvic fin, Anal fin, Caudal fin).
Data source location	Head Qadirabad, Phalia, Pakistan, University of Gujrat, Department of Computer Science.
Data accessibility	Repository name: [Mendeley data repository] Data identification number: [10.17632/n3ydw29sbz.3] Direct URL to data: [https://data.mendeley.com/datasets/n3ydw29sbz/3]

**Table:** 

**Value of the Data** • This is the sole dataset holding exact fish species identification based on six distinct classes i.e. *Ctenopharyngodon* *idella* (Grass carp), *Cyprinus carpio* (Common carp), Cirrhinus mrigala (Mori), *Labeo rohita* (Rohu), *Hypophthalmichthys molitrix* (Silver carp), and Catla catla (Thala). • Supports the testing of different classifiers based on the distinct features of fish species, and to compare their performance. • Gives information to evaluate algorithm performance under various fish species attributes such as shape and size of the body, Fins color and shape, eye, lateral line, head's size, and shape, scale size and shape. • In fisheries management, this dataset can be utilized to segregate species of fishes for the examination of underwater ecology and fish behavior. • A complex multiclass image dataset for the researchers to test several feature extractor and record their performance. • Data exerted in a controlled domain with a steady white background.

## 1 Data

Classifying fish can be valuable for various purposes one of which is the identification of different fish species. Classifying fish accurately are beneficial for the study of fish diversity [1]. Aside from this, the grouping of fishes is additionally valuable for learning the deportment and interspecies cooperation of fishes in a typical natural condition [2]. In the field of machine learning and computer vision, classification of fish species from images is a multi-class recognition issue and is an attractive research domain [3]. Automated fish classification is very important in fisheries research as it helps in automated monitoring of fish species activities in the ponds, feeding and diseases behavior.

**Fig. 1:**
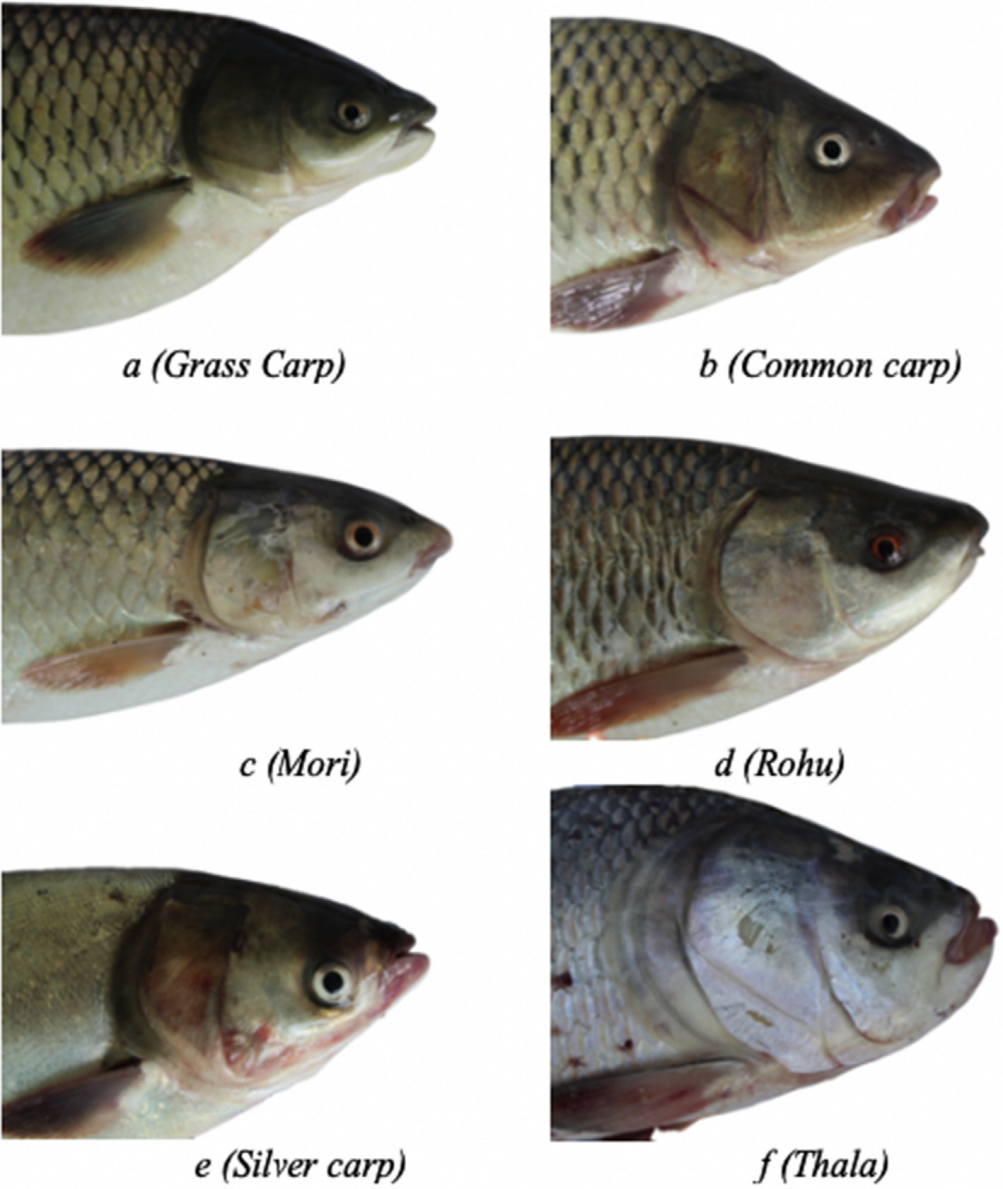
Example head images of 6 different fish species with transparent background taken from a particular position.

**Fig. 2:**
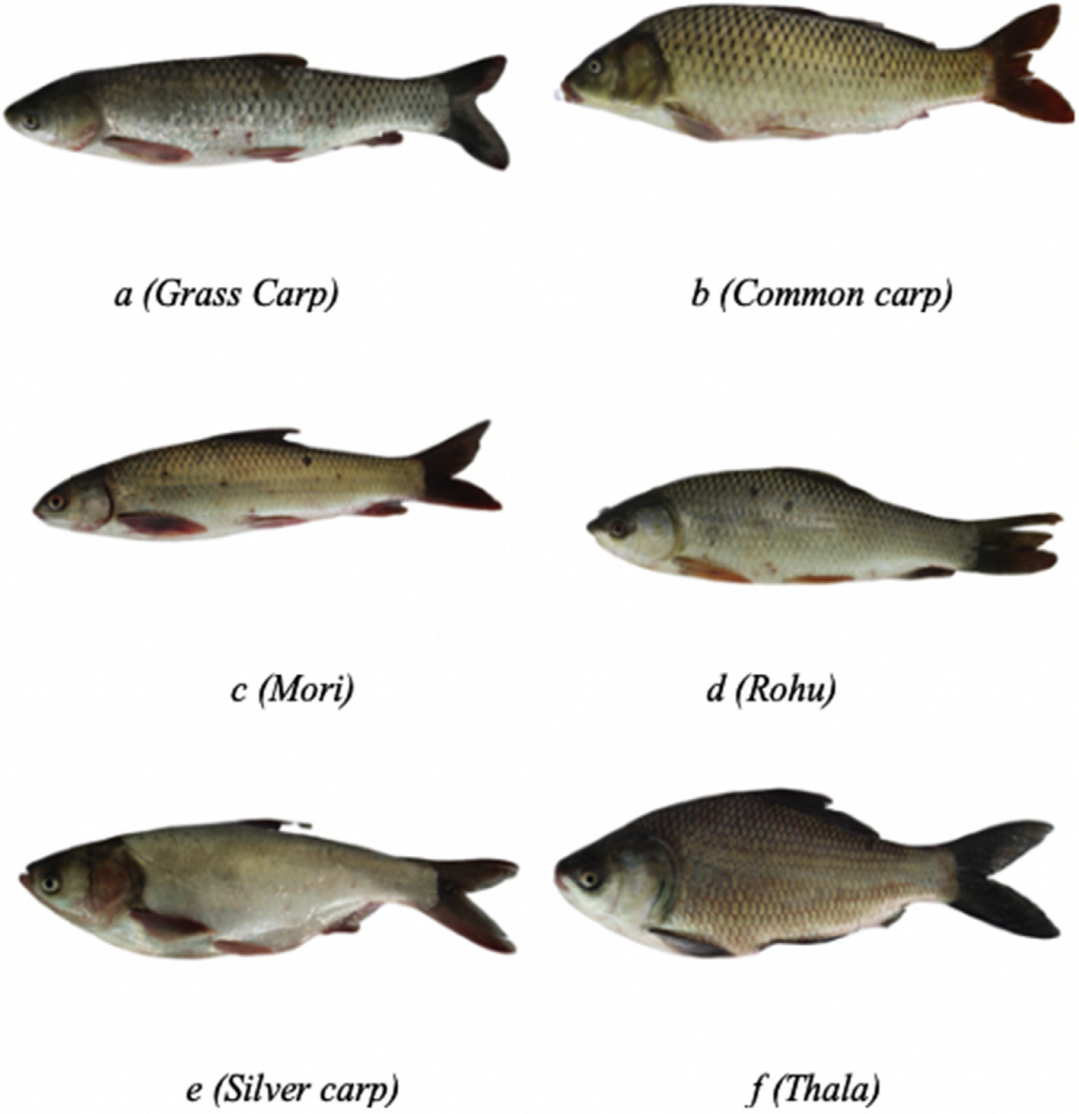
Example body images of 6 different fish species with transparent background taken from a particular position.

**Fig. 3:**
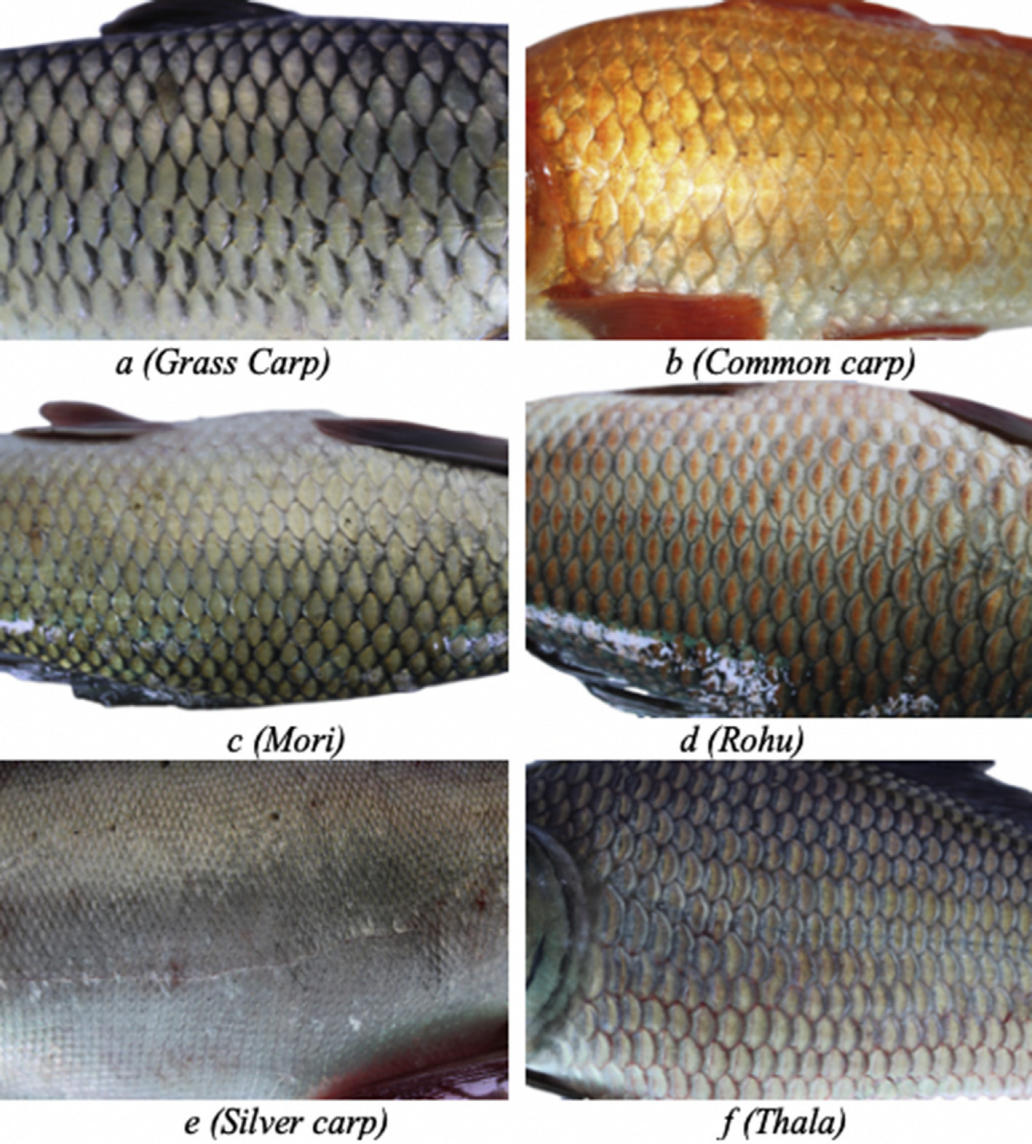
Example scale images of 6 different fish species with transparent background taken from a particular position.

Fish-Pak: a dataset of 6 different fish species i.e. *Ctenopharyngodon idella* (Grass carp), *Cyprinus carpio* (Common carp), Cirrhinus mrigala (Mori), *Labeo rohita* (Rohu), *Hypophthalmichthys molitrix* (Silver carp), and Catla (Thala), containing 915 images, popular for fish farming in the tropical areas of world including Pakistan. This dataset is type of multiclass containing 3 dominant features of fish species (Body, head and scale). Fish-pak dataset include 36 grass carp images of different poses (body, head, and scale) taken with the single camera. That 36 images of grass carp taken from the 5 different local farms located near head Qadirabad, Punjab. Similarly, Common carp (158 images), Mori (241 images) and Rohu (249 images) were captured manually from 3 different positions at the river of Chenab, Punjab, Pakistan. The remaining images of Fish-Pak dataset considering Silver carp (175 images) and Thala (56 images) were obtained from the Marala head works, Sialkot, Punjab with 3 different positions. All the images in Fish-Pak datasets are of fixed image dimension of 5184 × 3456 pixels and the resolution of 72 dpi. Fig. 1 shows the subset of head view images taken from the Fish-Pak randomly. Similarly Fig. 2, Fig. 3 contains complete body view and scale view of 12 different instances. We have preprocessed the data and make each image background transparent. Detailed characteristics with respect to different fish features of Fish-Pak dataset are given in Table 1.

**Table 1:** Morphological features of different fish species determined manually from Fish-Pak dataset.

Characters	*Labeo rohita* (Rohu)	*Cirrhinus mrigala* (Mori)	*Catla catla* (Thala)	*Hypophthalmichthys molitrix* (Silver carp)	*Cyprinus carpio* (Common carp)	*Ctenopharyngodon idella* (Grass carp)
Body shape	Spindle shaped	Elongated, streamlined or laterally compressed	Short and deep, somewhat laterally compressed	Deep and laterally compressed	Elongated, laterally compressed and back arched	Elongated, chubby and torpedo-shaped
Color	Blackish on the dorsal side and silvery on the ventro-lateral sides	Grayish or greenish on the back and silvery at the sides and below	Grayish on back and flanks, silvery-white at the below side	Greenish on the back, silvery on the belly	Silvery grey in with yellowish belly	Dark olive, shading to brownish-yellow on the sides with a white belly
Head region	**Mouth** is terminal.	**Mouth** is inferior.	**Mouth** is upturned.	**Mouth** is wide and slightly superior.	**Mouth** is large and slightly oblique.	**Mouth** is terminal to sub terminal.
	**Head** is equilateral.	**Head** is isosceles.	**Head** is broad.	**Head** is large and broad.	–	***Head*** is compressed and slightly pointed.
	**Snout** is depressed and projects beyond the jaws.	**Snout** is blunt.	**Snout** is bluntly rounded.	**Snout** is short and blunt.	**Snout** is long and blunt.	**Snout** is very short.
	**Lips:** Lower lip is fringed and folded. Extending upper lip which covers the lower lip.	**Lips:** Upper lip is entire and is not continuous with lower lip.	**Lips:** Upper lip is thin and covered by skin of snout. Lower lip is moderately thick.	–	**Lips** are thick with one pair of barbles on upper lip	**Lips** are non-fleshy and firm
Fin rays	**Dorsal fin** has 12–13 fin rays.	**Dorsal fin** has 12 to 13 rays.	**Dorsal fin** has18-19 rays.	**Dorsal fin** has 8 rays.	**Dorsal Fin** has 18–22 soft rays.	**Dorsal fin** has 7–8 rays.
	**Pectoral fin** has 17 rays.	–	–	–	**Pectoral fin** has 14–18 soft rays.	**Pectoral fin** has 15–20 rays.
	**Pelvic fin** has 9 rays.	–	–	–	**Pelvic fin** has 8 or 9 soft rays.	–
	**Anal fin** has 7 rays.	–	**Anal fin** has 8 rays.	**Anal fin** has 12 rays.	**Anal fin** has 4–6 soft rays.	**Anal fin** has 8–10 rays.
	**Caudal fin** has 19 rays.	–	–	**Caudal fin** has 21–22 rays.	**Caudal fin** has 19 soft rays	**–**

## 2 Experimental design, materials, and methods

### 2.1 Camera specification and setting

A digital camera (Canon EOS 1300D) with a sensor type of CMOS bearing the resolution of 5202 × 3465 (Mpix) and the sensor size of 14.9 × 22.3 (mm) was utilized for all image collection. The mode of camera was Scene, with the selection of sub-category as Snow scene, as it demonstrated the best mode for the unusual light condition of the case; with 14 megapixels picture measure (5184 × 3456 pixels) in 3:2 extents, glimmer and face discovery deactivated in the case when capturing fish body and scale. Furthermore, 2.5 instants zoom to all the more likely spot the fish head on the picture and less misuse of the image area with foundation. RGB shading space is selected for each of the images in JPG format, 8 pixels for each shading layer, adding 256 shades for every RGB layer.

### 2.2 Deep representation of feature maps

**Fig. 4:**
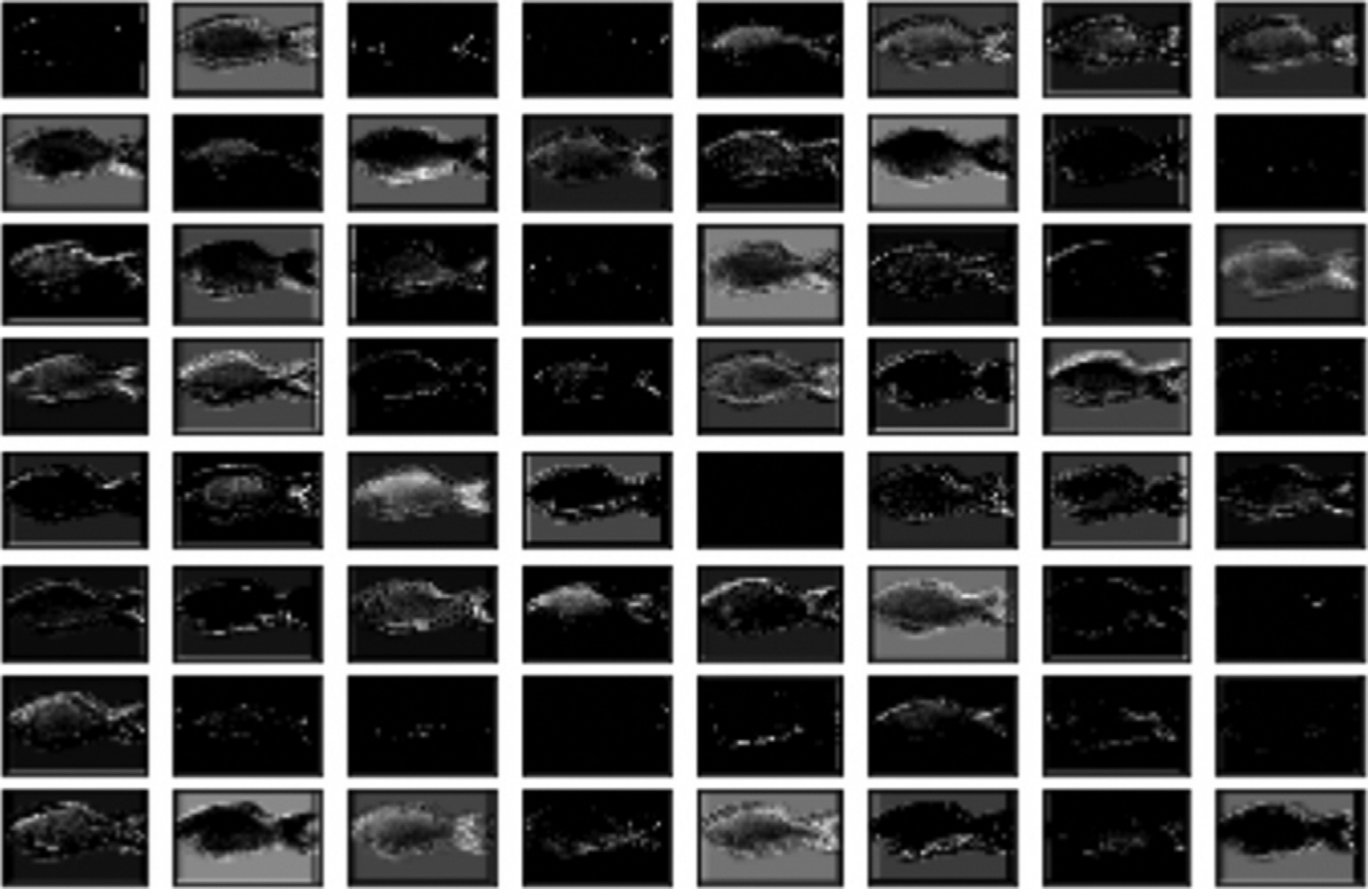
VGGNet feature map of 2nd convolutional layer with 64 maps and 3 × 3 kernel size.

We have applied CNN on the body images of Fish-Pak dataset and extract deep feature maps that can be found in Fig. 4. VGGNet [4] was selected to obtain the internal representation of the feature map from the 2nd convolutional layer with the first 64 maps. The kernel size for the experimentation was 3 × 3 with the pad of (1, 1) and stride of 128. We can see from Fig. 4, the more layer becomes deeper the more image becomes not interpretable by humans.

## Conflict of Interest

The authors declare that they have no known competing financial interests or personal relationships that could have appeared to influence the work reported in this paper.
